# Interaction of primary mast cells with *Borrelia burgdorferi* (*sensu stricto*): role in transmission and dissemination in C57BL/6 mice

**DOI:** 10.1186/s13071-017-2243-0

**Published:** 2017-06-27

**Authors:** Quentin Bernard, Zhenping Wang, Anna Di Nardo, Nathalie Boulanger

**Affiliations:** 1 EA7290 Virulence bactérienne précoce: groupe borréliose de Lyme, Fédération de médecine translationnelle et Faculté de Pharmacie de Strasbourg, Université de Strasbourg, Strasbourg, France; 2 Department of Dermatology, University of California, San Diego, USA; 3 Centre National de Reference Borrelia, Centre Hospitalier Universitaire, Strasbourg, France; 4 Present address: Department of Veterinary Medicine, University of Maryland, College Park, USA

**Keywords:** Mast cells, *Borrelia*, *Ixodes* tick, Pathogen transmission, *Kit*^*wsh−/−*^ mouse, Tick saliva

## Abstract

**Background:**

*Borrelia burgdorferi* (*sensu* *lato*), the causative agent of Lyme borreliosis is a bacterium transmitted by hard ticks, *Ixodes* spp. Bacteria are injected into the host skin during the tick blood meal with tick saliva. There, *Borrelia* and saliva interact together with skin cells such as keratinocytes, fibroblasts, mast cells and other specific immune cells before disseminating to target organs.

**Methods:**

To study the role of mast cells in the transmission of Lyme borreliosis, we isolated mouse primary mast cells from bone marrow and incubated them in the presence of *Borrelia burgdorferi* (*sensu stricto*) and tick salivary gland extract. We further analyzed their potential role in vivo, in a mouse model of deficient in mast cells (*Kit* ^*wsh−/−*^ mice).

**Results:**

To our knowledge, we report here for the first time the bacteria ability to induce the inflammatory response of mouse primary mast cells. We show that OspC, a major surface lipoprotein involved in the early transmission of *Borrelia*, induces the degranulation of primary mast cells but has a limited effect on the overall inflammatory response of these cells. In contrast, whole bacteria have an opposite effect. We also show that mast cell activation is significantly inhibited by tick salivary gland extract. Finally, we demonstrate that mast cells are likely not the only host cells involved in the early transmission and dissemination of *Borrelia* since the use of mast cell deficient *Kit* ^*wsh−/−*^ mice shows a limited impact on these two processes in the context of this mouse genetic background.

**Conclusions:**

The absence of mast cells did not change the replication rate of *Borrelia* in the skin. However, in the absence of mast cells, *Borrelia* dissemination to the joints was faster. Mast cells do not control skin bacterial proliferation during primary infection and the establishment of the primary infection, as shown in the C57BL/6 mouse model studied. Nevertheless, the *Borrelia* induced cytotokine modulation on mast cells might be involved in long term and/or repeated infections and protect from Lyme borreliosis due to the development of a hypersensitivity to tick saliva.

**Electronic supplementary material:**

The online version of this article (doi:10.1186/s13071-017-2243-0) contains supplementary material, which is available to authorized users.

## Background

*Ixodes* ticks are vectors of several pathogens including viruses (e.g. tick-borne encephalitatis virus), parasites (e.g. *Babesia*) and bacteria (e.g. *Borrelia*) [1]. They are transmitted to the host during the tick blood meal which lasts several days. Pathogens are inoculated into the skin with tick saliva. In the case of Lyme borreliosis, caused by *B. burgdorferi* (*sensu lato*) [2], bacteria first multiply in the skin before disseminating to target organs (heart, distant skin, joints) [3]. However, part of them persist for several weeks in the skin after transmission [4]. Skin cells, including keratinocytes, fibroblasts, endothelial cells, dendritic/Langerhans cells, T cells and mast cells (MCs), are those of immune importance since they are likely to be the first cells encountered by the bacteria [5]. Most of these cell functions have been shown to be inhibited by tick saliva compounds such as sialostatin L and Salp15 during bacteria transmission by *I. scapularis* ticks [6–12]. Notably, Salp15 protects *B. burgdorferi* (*s.l*.) from the host immune system by binding to the bacteria surface lipoprotein OspC [13]. OspC is a major *Borrelia* surface lipoprotein upregulated during the early transmission from the tick to the vertebrate host [14]. Moreover, Salp15 targets immune cells such as dendritic cells and T cells by inhibiting cytokine expression and cell maturation [15, 16]. The tick saliva also inhibits other mechanisms such as hemostasis, pain and itch to increase tick blood meal efficiency [17]. Pathogens have evolved to use these saliva-associated inhibition mechanisms to increase their transmission rate [11].

MCs are widely distributed throughout the body with specific locations at the surface epithelia such as the skin, the lung and gastrointestinal and genitourinary tracts [18]. These cells are well known for their association with pathological conditions such as asthma, allergy or anaphylaxis. MCs are increasingly studied with new evidence of their involvement in parasitic, bacterial and viral infections. They can directly sense Pathogen-Associated Molecular Patterns (PAMPs) through Pattern Recognition Receptors (PRRs) such as Toll Like Receptors (TLRs) [19]. They also can detect antigens through the binding of antibodies to their Fc receptors. MC response to pathogens depends on the PAMPs: some PAMPs induce only cytokine expression (TNF-α, IL-6, IL-13, IL-1β, IL-4, IL-5, IFN-γ) while others induce cytokine production as well as degranulation, a mechanism inducing preformed inflammatory mediator release [20]. In case of bacterial pathogenesis, the release of TNF-α, histamine or vascular endothelial growth factor (VEGF) by MCs increase vascular permeability. Chemokines produced by MCs increase the inflammatory cell attraction at the infection sites (eosinophils, neutrophils, NK cells) [21]. MCs can also directly kill pathogens through antimicrobial peptides such as cathelicidins [22]. Moreover, their ability to present antigen and to secrete inflammatory mediators allows the recruitment of dendritic cells as well as T cells at the infection site. All these processes give them a central role between innate and adaptive immunity [23, 24].

MCs have been poorly studied in the context of Lyme borreliosis. Currently, it is known that murine MC lines respond to *Borrelia* by secreting TNF-α and by slowly degranulating. This activity seems to depend in part on surface proteins but not on the lipoprotein OspA [25]. Fc receptors seem to be involved through an antibody-independent mechanism [26]. However, MC lines are not fully mature cells compared to bone marrow derived primary mast cells. In this study, for the first time we analyzed primary MCs response to *B. burgdorferi* (*sensu stricto*) and its lipoprotein OspC, a lipoprotein essential for the early bacterial transmission from the tick to the vertebrate host [27, 28]. We also explored in vitro the tick saliva impact on MCs response to *Borrelia*. Finally, we used a murine MC deficient model to study their involvement in the in vivo infection process.

## Methods

### *Borrelia*

*Borrelia burgdorferi* (*s.s*.) strain 297 was cultured in BSK-H medium (Sigma-Aldrich, St. Quentin Fallavier, France) at 33 °C, used at passage 8 in the late-log-phase, centrifuged, and then washed twice (30 min, 5000×*g*) with cell culture media. *Borrelia burgdorferi* (*s.s*.) 297, OspC-deficient and complemented mutants, have been described previously [14].

### Mast cell culture

Primary MCs were generated by extracting bone marrow cells from the femurs of 4 to 8 week-old C57BL/6 mice and culturing the cells at 37 °C, 5% CO_2_ in RPMI 1640 (Life Technologies, Villebon sur Yvette, France) supplemented with 10% inactivated FBS (Life Technologies), glutamine, non-essential amino acid (NEAA), penicillin/streptomycin, and 2-mercaptoethanol. Recombinant murine IL-3 (1 ng/ml; R&D Systems, Lille, France) and recombinant murine stem cell factor (SCF) (20 ng/ml; R&D Systems) were used in vitro for differentiation of the MC precursor [29]. Medium was changed twice a week. After 4 weeks, MC maturation and purity were confirmed by the expression of CD117 and FcεRI measured by flow cytometry and by metachromatic staining with toluidine blue. The purity of MCs was greater than 85% (Additional file 1: Figure S1).

### Mast cell degranulation and activation

Degranulation was assessed by measuring the activity of β-hexosaminidase [30–32] in the supernatants of 1 × 10^5^ MCs in 200 μl Tyrode’s buffer (0.1% bovine serum albumin, 0.1% glucose, 2 mmol/l MgCl_2_, 137.5 mmol/l NaCl, 12 mmol/L NaHCO_3_, 2.6 mmol/l KCl, pH 7.4) incubated for 2 h at 37 °C with different concentrations of *B. burgdorferi* (*s.s*.) (MOI 50:1 or 100:1), L-OspC (10 or 50 ng/ml), unlipidated-OspC (10 or 50 ng/ml) and/or saliva (20 μg/ml). For each sample assayed, supernatant aliquots (20 μl) were mixed with substrate solution (100 μl), which consisted of 1 mM 4-methylumbelliferyl-2-acetamide-2-deoxy-β-D-glucopyranoside (Calbiochem, Fontenay sous Bois, France) in 0.1 M sodium citrate buffer (pH 4.5), and were incubated for 2 h at 37 °C. The reaction was then stopped by the addition of 12 μl of 0.2 M glycine (pH 10.7). The reaction mixtures were excited at 365 nm and measured at 460 nm in a fluorescence plate reader (Gemini EM microplate spectrofluorometer, Molecular Devices). To determine the total cellular content of this enzyme, an equivalent number of cells were lysed with 1% triton X-100 (Sigma-Aldrich) and the plate blank was deducted. Release of β-hexosaminidase was calculated as the percentage of the total enzyme content.

Inflammatory response was assessed by incubating MCs with different concentrations of *B. burgdorferi* (*s.s*.) (MOI 50:1 or 100:1), L-OspC (10 or 50 ng/ml), unlipidated-OspC (10 or 50 ng/ml) and/or SGE (20 μg/ml) for 6 or 24 h at 37 °C. Cells and supernatants were collected and separated by centrifugation (300 g, 10 min).

### Tick salivary glands

SGE was obtained after dissection of female ticks fed for three days, and used at 20 μg/ml as described previously [7]. SGE was tested by the Limulus assay to check for the presence of endotoxins and was found to contain < 0.3 endotoxin units.

### Mice and bacterial challenge

MC-deficient (*Kit* ^*Wsh−/−*^) mice were kindly provided by Dr. Mécheri (Pasteur Institute, Paris, France). *Kit* ^*Wsh−/−*^ and C57BL/6 mice were bred at the animal facilities of the institute of bacteriology (University of Strasbourg, France) according to regulations of the CREMEAS. *Borrelia* (10^3^/100 μl) were intradermally injected into the dorsal thoracic area of mice. With a biopsy punch (Stiefel laboratory), 3 mm skin biopsies were then collected at 3, 5, 7 and 15 days after the inoculation for *Borrelia* quantification by quantitative PCR (qPCR). Quantification targeting the *flab* gene was performed as described previously [33]. Numbers of *Borrelia* in the skin were normalized to 10^4^ GAPDH DNA copies. Organs were collected at different time points post infection to check for the presence of spirochetes, either by culture in BSK-H medium and observation under dark field microscopy (heart, joint) or by PCR (ear tissue).

### ELISA

To measure IL-6 secreted by MC, enzyme-linked immunosorbent assays (ELISAs) were performed on cell supernatants. Protocols were based on sandwich techniques, as described by the manufacturer (R&D systems, Lille, France).

### Flow cytometry

MCs were centrifuged at 800× *g* for 15 min at room temperature. Cells (5.10^5^ cells/ml) were resuspended in PBS-EDTA 3 mM. Fc receptors were blocked with Fc blocking reagent (CD16/CD32) for 5 min. Cells were finally processed for staining with PE-labeled rat anti-mouse CD 117 (eBioscience, Fontenay sous Bois, France), (1/300) and APC-labeled american hamster anti-mouse FcεRI (eBioscience, 1/300) in PBS-EDTA 3 mM before analysis with a FACScalibur (BD Biosciences, Le Pont de Claix, France) equipped with Flowjo software.

### Real-time quantitative RT-PCR

Quick-RNA MiniPrep (Zymo Research, St Quentin En Yvelines, France) was used to isolate total RNA. One microgram of total RNA for cDNA synthesis by the iScript cDNA Synthesis Kit (Bio-Rad, Marnes la Coquette, France) was used; it was amplified by real-time RT-PCR in an ABI 7300 Real-Time PCR system (Applied Biosystems, Illkirch, France). The amplification cycle consisted of initial denaturation at 95 °C for 10 min followed by 45 cycles each at 95 °C for 10 s and 60 °C for 1 min and a final melt curve analysis: 55 °C for 30 s with an increase of 0.5 °C/cycle to 95 °C. Primers, probes and RNA analysis reagents (TaqMan Master Mix reagents kit) used for real-time RT-PCR were obtained from Applied Biosystems (Illkirch, France). We used the comparative ΔΔC_T_ method to determine the quantification of gene expression, normalized the target gene (IL-6, F: 5′-TAG TCC TTC CTA CCC CAA TTT CC-3′ and R: 5′-TTG GTC CTT AGC CAC TCC TTC-3′; MCP-1, F: 5′-CTT CTG GGC CTG CTG TTC-3′ and R: 5′-CCA GCC TAC TCA TTG GGA TCA-3′; FlaB, F: 5′-TTT CAG GGT CTC AAG CGT CCT G-3′ and R: 5′-GCA GGT GCT GGC TGT TGA GC-3′) expression in the test samples to the endogenous reference GAPDH (F: 5′-CCA ACC GCG AGA AGA TGA CC-3′ and R: 5′-GAT CTT CAT GAG GTA GTC AGT-3′) level and reported them as the fold difference relative to GAPDH gene expression in untreated baseline control. We performed all the assays in triplicate and repeated the experiments at least three times.

### Statistical analyses

Each experiment was carried out at least three times in independent experiments. The most representative experiment is shown. Results represent the mean ± standard deviation (SD) of at least triplicates of one experiment and were analyzed by two-tailed Student’s *t*-test or ANOVA with Tukey *post-hoc* test using GraphPad software. Differences in values were considered significant if *P* < 0.05 (*P < 0.05; **P < 0.01; ***P < 0.001). For in vivo studies, a minimum of 5 mice has been used at each time point of the kinetics to get significant results.

## Results

### *Borrelia burgdorferi* (*s.s*.) 297 and its lipoprotein L-OspC differently activate mast cells

**Fig. 1:**
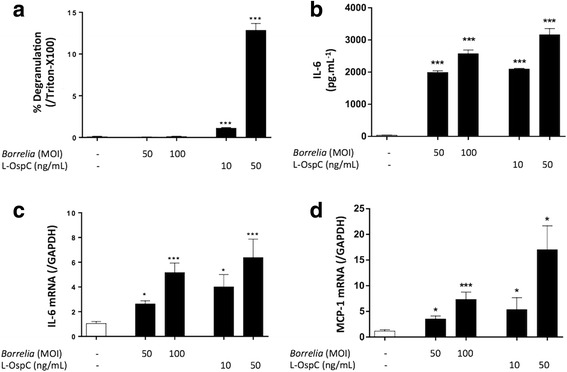
Mouse mast cell response to *Borrelia* and OspC. Primary MCs from C57BL/6 mice were activated with different concentrations of *Borrelia burgdorferi* (*s.s*.) 297 strain (MOI 10:1 or 50:1) or Lipidated-OspC (L-OspC: 10 or 50 ng/ml). After 2 h, the level of degranulation was evaluated by measuring β-hexasominidase release and percentage degranulation was calculated according to the negative control (**a**). After 24 h, supernatants were collected to measure IL-6 concentration by ELISA (**b**). After 6 h, RNAs were collected to measure IL-6 and MCP-1 mRNA expression (**c**, **d**). *P < 0.05; ***P < 0.001

Primary MCs generated by culturing mice bone marrow cells for 4 weeks with IL-3/SCF were used. To study the impact of *Borrelia* on MC activation, primary MCs were incubated with different concentrations of *B. burgdorferi* (*s.s*.) 297 or its surface lipoprotein OspC (Fig. 1). Degranulation was only induced by L-OspC, not by whole *B. burgdorferi* (*s.s*.) 297 (Fig. 1a) or by unlipidated OspC protein (data not shown). However, both L-OspC and *Borrelia* induced pro-inflammatory cytokine and chemokine expression by MC at protein (IL-6) and mRNA (IL-6 and MCP-1) levels (Fig. 1b-d). Cathelicidin, an antimicrobial peptide well-known to be produced by MCs [22], was poorly or not induced in our experiment (data not shown).

### L-OspC is not essential for murine mast cell activation by *Borrelia*

**Fig. 2:**
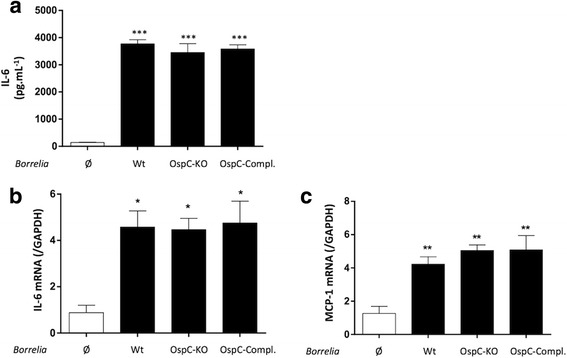
Mouse mast cell response to *Borrelia* OspC-KO. Primary MCs from C57BL/6 mice were activated with medium (Ø) *Borrelia burgdorferi* (*s.s*.) (Bb) 297 (Wt), Bb OspC-KO (OspC-KO) or Bb OspC-complemented strains (OspC-Compl.) (MOI 50:1). After 24 h, supernatants were collected to measure IL-6 concentration by ELISA (**a**). After 6 h RNAs were collected to measure IL-6 and MCP-1 mRNA expression (**b**, **c**). *P < 0.05; **P < 0.01; ***P < 0.001

OspC is an essential lipoprotein for the transmission of *Borrelia* from the tick to the vertebrate host [27, 28]. However, to determine whether L-OspC is essential for MC activation in vitro in the context of the whole bacteria, we activated primary MC with an OspC-KO mutant strain of *B. burgdorferi* (*s.s*.) 297 (Fig. 2). We show here that IL-6 protein secretion as well as IL-6 and MCP-1 mRNA expression were similarly induced by wild type, mutant and the complemented strains.

### Mast cell inflammatory response against *Borrelia* is inhibited in part by tick saliva

**Fig. 3:**
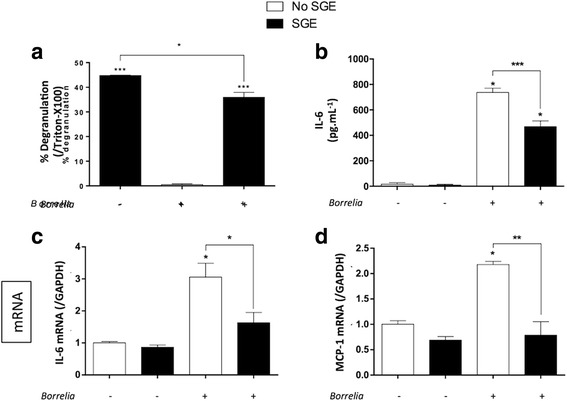
Impact of tick saliva on mouse mast cell inflammatory response*.* Primary MCs from C57BL/6 mice were activated with *Borrelia burgdorferi* (*s.s*.) 297 strain (MOI 50:1) in the absence (*white* bars) or presence (*black* bars) of tick salivary gland extract (SGE: 20 μg/ml). After 2 h, the level of degranulation was evaluated by measuring β-hexasominidase release and percentage degranulation was calculated (**a**). After 24 h, supernatants were collected to measure IL-6 concentration by ELISA (**b**). After 6 h, RNAs were collected to measure IL-6 and MCP-1 mRNA expression (**c**, **d**). *P < 0.05; **P < 0.01; ***P < 0.001

Since *B. burgdorferi* (*s.s*.) 297 transmission occurs in the presence of tick saliva, we explored its effect on the MC inflammatory response in vitro (Fig. 3). Tick salivary gland extract (SGE) was incubated with primary MCs in the presence or absence of *B. burgdorferi* (*s.s*.) 297. Tick SGE induces mast cell degranulation, but MC-degranulation induced by tick SGE is reduced in the presence of *Borrelia* (Fig. 3a). Moreover, tick SGE significantly inhibits the IL-6 (t-test: *t*_(4)_ = 7.03, *P* = 0.002 for mRNA, t-test: *t*_(5)_ = 9.74, *P* = 0.0002 for protein) and MCP-1 (t-test: *t*_(5)_ = 2.93, *P* = 0.032) expression induced by *Borrelia*-activated MC (Fig. 3b–d).

### Mast cells do not impact *B. burgdorferi* (*s.s*.) 297 amplification and dissemination during primary infection

**Fig. 4:**
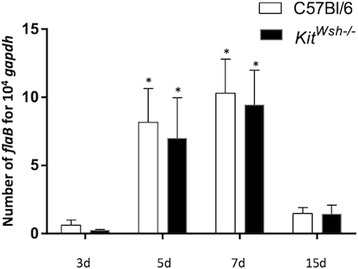
Role of mast cells upon mouse infection by *Borrelia*. C57BL/6 (*white* bars) or Kit^Wsh −/−^ (*black* bars) mice were infected intradermally with *Borrelia burgdorferi* (*s.s*.) (Bb) 297 strain (10^3^ bacteria/100 μl) by syringe inoculation. The relative quantification of Bb, presented as copies of *flab* transcript per 10^4^ copies of *gapdh* transcripts, at the site of inoculation (skin of the mouse back) was measured by qPCR at different time points (d = day) after the infection. Results of three separate experiments are presented. *P < 0.05

We have seen that *B. burgdorferi* (*s.s*.) 297 activates primary MCs in vitro in an L-OspC-independent way. However, the role of MC in vivo during *B. burgdorferi* (*s.s*.) 297 infection has never been investigated so far. To explore the impact of MC during mouse infection by *B. burgdorferi* (*s.s*.) 297*,* we analyzed bacteria multiplication in the skin as well as its dissemination in wild type C57BL/6 *versus* *Kit* ^*Wsh−/−*^ mice (Fig. 4, Table 1). No significant difference was observed in terms of bacteria multiplication in the skin (ANOVA: *F*_(3, 65)_ = 0.43, *P* = 0.72) (Fig. 4). *Borrelia* dissemination in mice was not significantly altered in the absence of mast cells, although one *Kit* ^*Wsh−/−*^ mouse showed a positive *Borrelia* count at day 15 at the ear while all C57BL/6 mice were negative (Table 1).

**Table 1:** Impact of mast cell deficiency on *B. burgdorferi* (*s.s*.) 297 dissemination

Day/ Organ	Joints (%)^a^	Heart (%)^a^	Ear (%)^a^
C57BL/6 (10 weeks)			
Day 3 (*n* = 8)	0	0	0
Day 5 (*n* = 8)	0	0	0
Day 7 (*n* = 5)	60	0	0
Day 15 (*n* = 8)	75	0	0
*Kit* ^*Wsh −/−*^ (10 weeks)			
Day 3 (*n* = 7)	0	0	0
Day 5 (*n* = 10)	10	0	0
Day 7 (*n* = 12)	50	0	0
Day 15 (*n* = 10)	90	0	10

Wild type or Kit^Wsh −/−^ mice were infected intradermally with *Borrelia burgdorferi* 297 strain (10^3^ bacteria/100 μl) by syringe inoculation. The dissemination of *Borrelia* was determined by in vitro culture and by qPCR performed on the different distant organs: the joint, the heart and the ear

^a^The percentage of positive samples to *Borrelia* is calculated according to 5 mice minimum per group

*Abbreviations*: *n* number of mice per group, *day* day of infection

## Discussion

The skin is one of the first defense organ that pathogens have to cross to infect the host in the context of arthropod-borne diseases [5, 34]. Some bacteria, parasites and viruses have evolved to use vectors, such as ticks and mosquitoes, to bypass this physical barrier. However, once pathogens have been injected into the skin by vectors, they have to face a biological barrier made of skin cells and inflammatory molecules. In Lyme borreliosis, bacteria remain in the skin for days, first interacting with resident skin cells and immune cells before disseminating to target organs (joints, ear, heart, nervous system). Those cells, including keratinocytes, fibroblasts, macrophages, dendritic cells, neutrophils, NK cells, T and B cells secrete inflammatory molecules such as antimicrobial peptides, cytokines, chemokines, adhesins and reactive oxygen species to stop the infection [31–42]. MCs are present in the skin in large quantity. Early studies on the interaction of MCs with *B. burgdorferi* (*s.s*.) have been done with rat peritoneal MCs and murine MC/9 MCs line [25, 26], and, to our knowledge, the ability of mice primary MCs to interact with *Borrelia* has not been investigated to date. Cell lines are widely used in many types of cancer research as well as in immunological or metabolism studies. However, their behavior may differ from primary cells [42]. We thus decided to explore the ability of mouse primary MCs to respond to *B. burgdorferi* (*s.s*.) 297. Primary MCs express IL-6 and MCP-1 in response to *Borrelia*. The expression of these two genes has been shown in mouse skin in response to *Borrelia* infection [3]*.* Moreover, IL-6 has been essentially associated with late symptoms such as arthritis and neuroborreliosis [43, 44], but also with erythema migrans and with late skin symptoms [45]. IL-6 is of importance since this cytokine has a crucial role during the transition from innate to acquired immunity. Its potential release in the skin by resident MCs might participate in early cell attraction such as neutrophils [46]. MCs also express IL-6 and MCP-1 in response to L-OspC. OspC is an essential lipoprotein when it enters the host skin and is already expressed by the bacteria during the tick blood meal [47]. If OspC role is not clear, its involvement in the early course of the infection has been demonstrated [28] and recent data [48] tend to indicate that it has an antiphagocytic activity. Also, OspC exhibits a protective role against the innate immune system [49]. A previous study has determined the inability of the lipoprotein OspA to activate TNF-α secretion by MC/9 MCs [25]. OspA is mainly expressed in ticks and is downregulated during the early transmission to the vertebrate hosts [50]. MC activation thus differs between *Borrelia* lipoproteins, since the lipidated OspC protein induced a strong degranulation, but not OspA nor the unlipidated OspC protein. Moreover, although lipoproteins are the major molecules on the surface of the bacteria [51], whole *Borrelia* parasites do not induce mast cell degranulation of mast cell line [25] and primary mast cells. Altogether, we can assume that the lipid part of OspC, anchored and hidden in the bacteria membranes, induces degranulation. Moreover, the amount of OspC present on the whole bacteria might be not sufficient to induce degranulation compared to the purified lipoprotein. Globally, the differences might be due to the cell state (line *versus* primary) or molecules studied (TNF-α *versus* IL-6/MCP-1). However, OspC is not sufficient and the only lipoprotein involved in MC activation in the context of the whole bacteria since an OspC-deficient strain of *B. burgdorferi* (*s.s*.) 297 still activates MC similarly to the wild type. *Borrelia* is recognized by different Toll like receptors (TLRs) [52] and the absence of OspC might be counteracted by other *Borrelia* surface antigens like flagellin interacting with TLR5. It might also be balanced by the presence of other lipoproteins or even other ligands since *B. burgdorferi* (*s.s*.) 297 activate immune cells through many PRRs [53]. Our experiment also suggests that in contrast to lipopolysaccharides or lipotechoic acid [54], *Borrelia* and its lipoprotein are not able to induce cathelicidin expression by MCs. This might be due to the different TLRs those ligands are associated with.

During *Borrelia* transmission to the vertebrate host, bacteria are injected into the skin with tick saliva. We have shown here that tick salivary gland extract significantly but not completely inhibits IL-6 and MCP-1 expression by MCs. The ability of tick saliva to decrease inflammatory cytokine secretion was already observed on other cell types such as keratinocytes, dendritic cells and T cells. To determine whether the inhibition is due to a direct binding of salivary molecules to the bacteria, or to the cells, requires further studies, given the complexity of the tick saliva [11]. The impact of tick saliva on MCs during *B. burgdorferi* (*s.s*.) 297 transmission has not been investigated before. However, the involvement of MCs during non-infectious tick bite was analyzed in several studies. Histological analyses of BALB/c mice skin after *Dermacentor variabilis* tick bite, revealed an increased number of MCs especially after second or third tick infestation [55, 56]. Similar observations were made with *Ixodes ricinus* infested rabbits [57]. However, the role of MCs in acquired tick resistance seems to depend on the tick species involved [56, 58]. Although tick saliva induces MC degranulation, it is also known to contain molecules able to counteract granules-contained mediators after their release. For instance, tick saliva contains lipocalins which can bind histamine to inhibit their functions, especially itching feelings [59]. Interestingly, we have seen that *B. burgdorferi* inhibits in part the MCs degranulation induced by tick saliva. This mechanism might help the bacteria to reduce inflammation. Similar observation was made with another bacteria, a non-pathogenic *Escherichia coli* strain, able to inhibit anti-2,4 dinitrophenol IgE sensitized MCs degranulation [60]. However, it remains to be explored if the *B. burdorferi* mediated inhibition of the degranulation induced by tick saliva is specific to this spirochete or not.

*Borrelia burgdorferi* (*s.s*.) multiplies first in the skin of C3H/HeN mouse before to efficiently disseminate to target organs such as the heart, the brain or the joints [33]. We thus explored the impact of MCs deficiency on the multiplication and the dissemination of the bacteria using a MC deficient murine model. This model already revealed an important role of MCs during *Leishmania* infection, a vector-borne disease transmitted by *Phlebotomus* sand flies [61, 62]. Since in Lyme borreliosis, the role of MCs has never been investigated in vivo, we infected MC deficient mice with *B. burgdorferi* (*s.s*.) 297 and compared to wild type mice, C57BL/6. Interestingly, the absence of MC does not influence bacteria multiplication happening at day 5/7 post infection in mice as previously described in C3H/HeN mice infected with *Borrelia* [3]. It does not alter significantly the bacteria dissemination ability neither. Despite the MC ability of this mouse strain to promote in vitro an inflammatory response against *Borrelia*, in our in vivo model, mast cells do not control bacterial proliferation during primary infection and the establishment of the primary infection. It is also known that the genetic background of MCs deficient mice (C57BL/6) is not optimal to study *B. burgdorferi* (*s.s*.) 297 infection since it shows some resistance [63]. MCs might have a higher impact on more susceptible mice such as C3H/HeN mice, but no MC deficient mice are available in this genetic background. In addition, we had to wait 10 weeks in Kit^Wsh −/−^ mice to get a very low level of MCs [64]. This age might also likely affect the outcome of infection since in C3H/HeN mice, the infection of mice with *Borrelia* is usually performed at 3 weeks of age. As with tick infestation [55, 56], a role of MCs could occur during secondary of tertiary infection by *B. burgdorferi* (*s.s*.) 297. Moreover, MC might have a role on long term infection, by stimulating the adaptive system [65].

## Conclusions

MCs have been associated to the disease severity in malaria, another vector-borne disease [66]. In Lyme disease, these cells appear to be of lesser importance against *Borrelia*. In our study, we show that MCs do not have a protective role against *Borrelia* infection in vivo. However, these cells might be important for repeated infections. In patients regularly bitten by ticks, a skin inflammatory response is clearly described [67, 68] with an IgE antibody response against tick saliva [69]. The impact of tick saliva resistance on a subsequent *Borrelia* infection remains to be explored in models such as guinea pigs, since mice do not really develop tick saliva resistance [70].

## Additional file

Additional file 1: Figure S1. Mast cell purity analyzed by flow cytometry. Primary MCs generated by culturing mice bone marrow cells for 4 weeks with IL-3/SCF were processed for staining with PE-labeled rat anti-mouse CD 117 and APC-labeled american hamster anti-mouse FcεRI. A FACScalibur equipped with Flowjo software was used for flow cytometry analysis. (TIFF 8219 kb)

## Abbreviations

ELISA: Enzyme-linked immunsorbent assay
IL: Interleukin
MC: Mast cell
NEAA: Non-essential amino acid
OI: Multiplicity of infection
Osps: Outer surface proteins
Pamp: Pathogen-associated molecular pattern
PRR: Pattern recognition receptor
qPCR: Quantitative polymerase chain reaction
SCF: Stem cell factor
SGE: Salivary gland extract
TLR: Toll-like receptor
