# Supplementary figures and images for: Interaction of primary mast cells with Borrelia burgdorferi (sensu stricto): role in transmission and dissemination in C57BL/6 mice
Source: Parasit Vectors. 2017 Jun 27;10:313. doi: 10.1186/s13071-017-2243-0 (PMC5488306; doi:10.1186/s13071-017-2243-0)

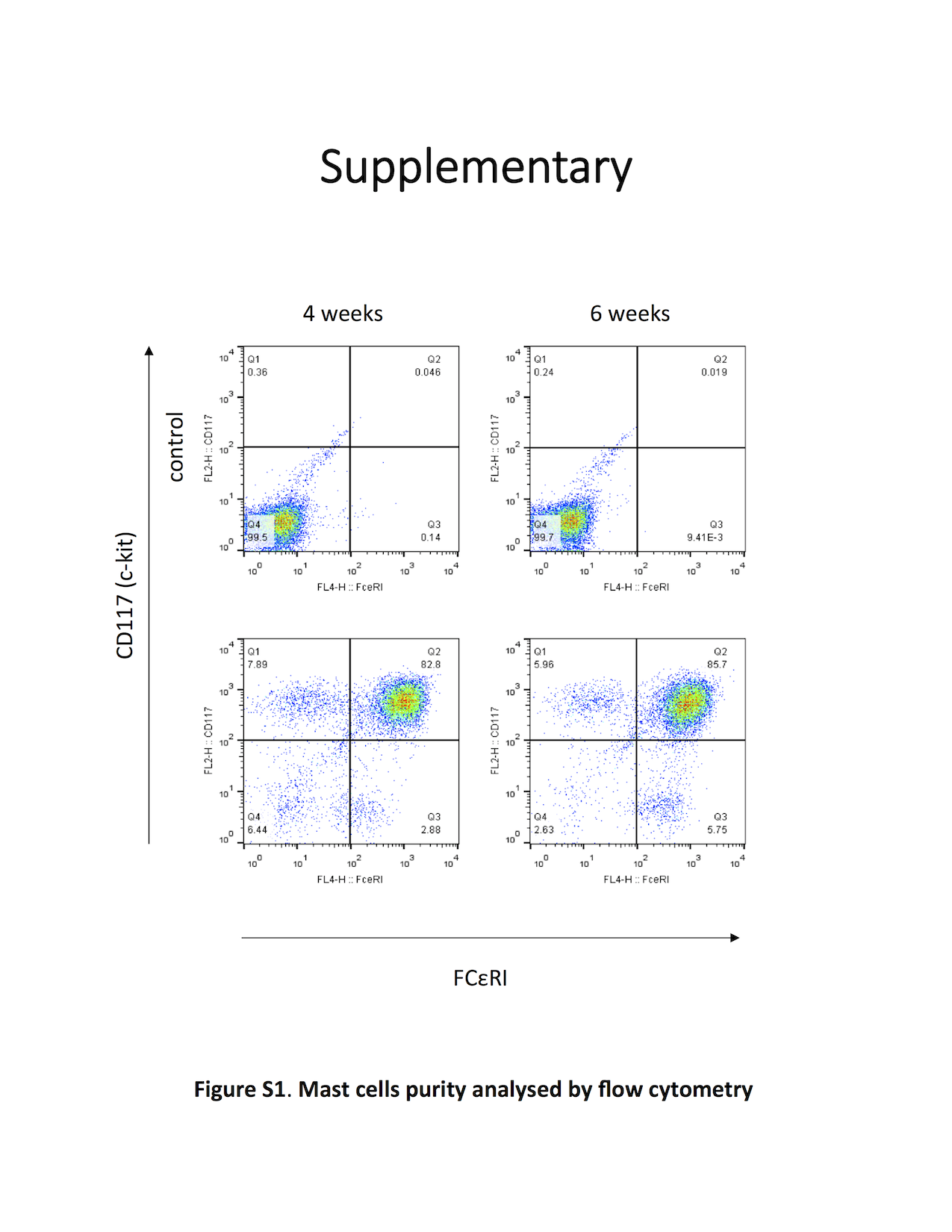

Supplement: Additional file 1: Figure S1. — Mast cell purity analyzed by flow cytometry. Primary MCs generated by culturing mice bone marrow cells for 4 weeks with IL-3/SCF were processed for staining with PE-labeled rat anti-mouse CD 117 and APC-labeled american hamster anti-mouse FcεRI. A FACScalibur equipped with Flowjo software was used for flow cytometry analysis. (TIFF 8219 kb) [file 13071_2017_2243_MOESM1_ESM.tiff]
